# From Colitis to Cancer: An Evolutionary Trajectory That Merges Maths and Biology

**DOI:** 10.3389/fimmu.2018.02368

**Published:** 2018-10-16

**Authors:** Ibrahim Al Bakir, Kit Curtius, Trevor A. Graham

**Affiliations:** ^1^Evolution and Cancer Laboratory, Centre for Tumour Biology, Barts Cancer Institute, London, United Kingdom; ^2^Inflammatory Bowel Disease Unit, St Mark's Hospital, Harrow, United Kingdom

**Keywords:** inflammatory bowel disease (IBD), colorectal cancer, cancer evolution, risk stratification, biomarker development, surveillance colonoscopy

## Abstract

Patients with inflammatory bowel disease have an increased risk of developing colorectal cancer, and this risk is related to disease duration, extent, and cumulative inflammation burden. Carcinogenesis follows the principles of Darwinian evolution, whereby somatic cells acquire genomic alterations that provide them with a survival and/or growth advantage. Colitis represents a unique situation whereby routine surveillance endoscopy provides a serendipitous opportunity to observe somatic evolution over space and time *in vivo* in a human organ. Moreover, somatic evolution in colitis is evolution in the ‘fast lane': the repeated rounds of inflammation and mucosal healing that are characteristic of the disease accelerate the evolutionary process and likely provide a strong selective pressure for inflammation-adapted phenotypic traits. In this review, we discuss the evolutionary dynamics of pre-neoplastic clones in colitis with a focus on how measuring their evolutionary trajectories could deliver a powerful way to predict future cancer occurrence. Measurements of somatic evolution require an interdisciplinary approach that combines quantitative measurement of the genotype, phenotype and the microenvironment of somatic cells–paying particular attention to spatial heterogeneity across the colon–together with mathematical modeling to interpret these data within an evolutionary framework. Here we take a practical approach in discussing how and why the different “evolutionary ingredients” can and should be measured, together with our viewpoint on subsequent translation into clinical practice. We highlight the open questions in the evolution of colitis-associated cancer as a stimulus for future work.

## Inflammatory bowel disease and colorectal cancer–the clinical background

Patients with inflammatory bowel disease (IBD) have an increased risk of developing colorectal cancer (CRC); this risk is related to disease duration, extent (1), and cumulative inflammation burden (2, 3). Patients with longstanding, extensive colonic inflammation develop colorectal cancer at a younger age compared to the general population, and are more likely to suffer from multifocal neoplasia. For this reason, across the world, patients are enrolled (4, 5) into surveillance programs that aim to detect early cancers and precursor dysplastic lesions with the hope of possible curative intervention before a symptomatic cancer develops. Current surveillance programs are plagued by oversurveillance and overtreatment of low-risk patients, as well as interval cancers (that is, symptomatic cancers detected in between surveillance colonoscopies) in high risk patients (6). These challenges reflect our poor understanding of the molecular processes underpinning colorectal carcinogenesis in IBD. We propose the use of an evolutionary approach combining genetic, environmental, immune and microbiome parameters to better stratify IBD patients by CRC risk.

## Cancer evolution–the theory of carcinogenesis

**Figure 1:**
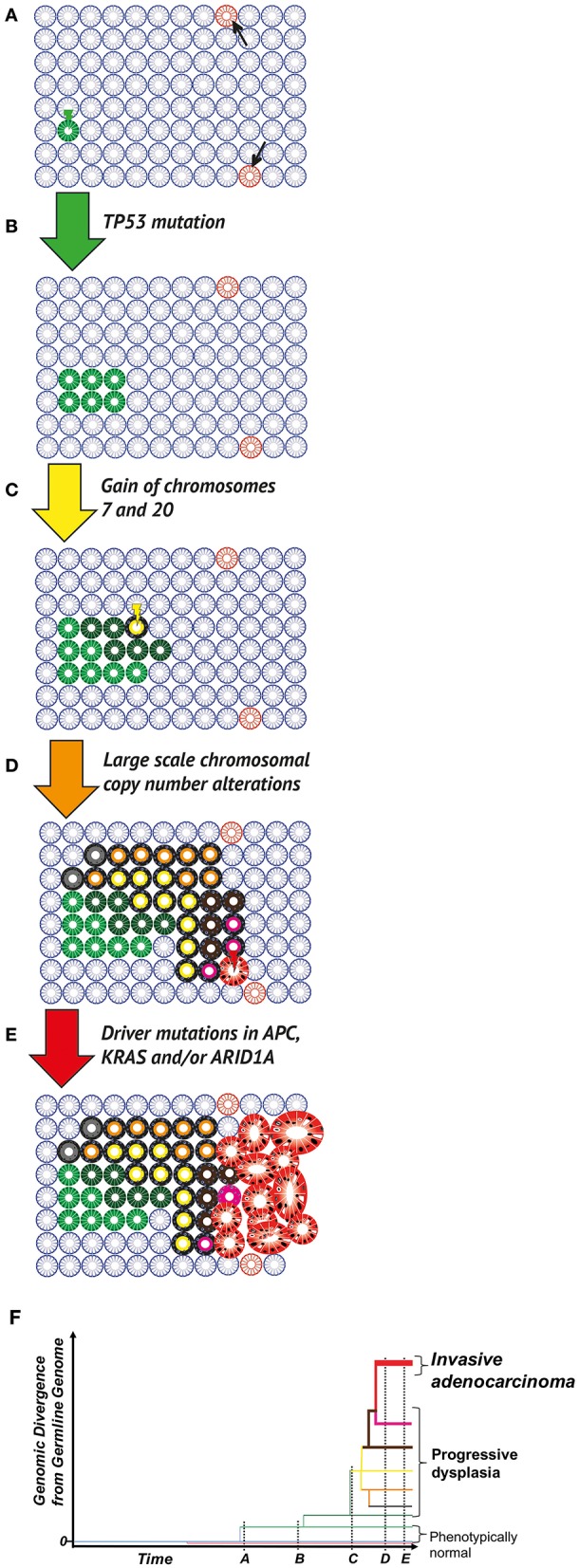
Clonal evolution in the colitic bowel. **(A)** The normal colon. Wild type (blue), phenotypically normal epithelial cells within crypts acquire mutations occur through chance (DNA replication error at mitosis) or due to microenvironmental pressures (e.g., mutagens in the diet or microbiome). Most mutations (pink crypts, arrows) are evolutionary neutral (i.e., do not change phenotype), or in fact disadvantageous leading to eventual cell death, through the disruption of critical cellular mechanisms or the activation of senescence/apoptosis. Occasionally, a positively selected driver mutation will be acquired. Through either positive selection or neutral drift, the mutant cell population can grow and take over the whole population of cells within the crypt (green crypt). **(B)** Field cancerization. A crypt wholly populated by mutated cells may have an increased crypt fission rate and/or lowered crypt death rate (green crypt). This leads to the expansion of the mutant crypt through the epithelium (green patch), causing a “field” of crypts bearing the same base change, heritable epigenetic change or copy number alteration, which is disposed to subsequent neoplastic transformation. **(C)** Neoplastic initiation. IBD generates a selective pressure favoring clones capable of surviving repeated cycles of inflammation and more rapidly repopulating the healing mucosa during remission. These selected mutant clones, while remaining histopathologically unremarkable, continue to evolve and expand at varying rates (crypts in various shades of green). Expansion remains limited by environmental constraints (e.g., a reliance on paracrine signaling from the stroma or a limit to the tolerance of cell crowding). Within this cancerised field, a frankly dysplastic sub-clone (yellow crypt) eventually arises. **(D)** Neoplastic promotion. The dysplastic clone (yellow with black outline) grows at a much more rapid pace than non-dysplastic crypts, resulting in an accelerated rate of evolution (crypts with black outline and shades of orange, brown, gray or pink); this genetic progression may be accompanied by progression in dysplasia grading. Potential biological mechanisms are diverse and include a loss of dependence on morphogen gradients and an altered metabolism that is better adapted to a hypoxic and nutrient-poor environment. **(E)** Malignant transformation and progression. Transformation to a malignant phenotype (red gland) produces a clone that can undergo uncontrolled cell division and is capable of invasion. Further progression in clone size results in a symptomatic, clinically detectable cancer. Potential biological mechanisms for this malignant transformation include a loss of critical DNA repair mechanisms and escape from immune surveillance. **(F)** Phylogenetic representation. Through multi-region sequencing, quantification of genomic divergence, and mathematical modeling, the phylogenetic relationship between clones can be understood. Here the evolutionary history of an IBD-CRC and its precursor lesions is depicted, with branch lengths corresponding to the amount of genetic change from the bifurcation node of clone branches.

The marked variability of cancers in terms of tissue origin, histopathological subtype, clinical progression and response to medical therapy, belies a shared mechanistic origin: namely, that all cancers are diseases caused by the acquisition of heritable genomic changes, which provide these mutant cells with a survival or growth advantage over their neighbors. During the stages of neoplastic initiation, promotion, progression and malignant conversion (Figure 1), the tissue microenvironment generates a (variable) selective pressure that defines those advantageous phenotypic traits (7, 8). In this respect, evolutionary principles, long utilized in the understanding a host of other biological phenomena, from the origin of new species to the emergence of drug-resistant pathogens, can also be applied to understand carcinogenesis (9). Measurements of the clonal composition of a neoplasm, namely the proportion of tumor cells bearing particular mutations, can be readily quantified and used to infer dynamics of the evolutionary process, including when clones emerged and how quickly they spread through the neoplasm (10).

## Evidence for large scale temporospatial clonal evolution in IBD

“Field cancerization” was first described over 50 years ago to explain the presence of large regions of histopathologically abnormal pre-cancerous cells in the oral mucosa from which multiple squamous cancers arose (11); that cancerised fields arise from the clonal expansion(s) of (epi)genetically altered cells was shown later [see reviews (12, 13)]. Field cancerization has been described in the premalignant colon, and we have previously postulated that field cancerization can occur either as a single large clonal expansion, or via the parallel expansion of multiple large clones (“clonal mosaicism”) (14). The two modes may co-occur, with small areas of mosaicism contained within a large expansion. One study analyzing the genomic abnormalities in individual crypts within different neoplastic lesions, demonstrated their monoclonality across most but not all lesions (15); in some lesions the adjacent phenotypically normal crypts shared the same driver mutation. Other studies have revealed field cancerization across large regions of the colon: Galandiuk et al. (16) undertook a chronological assessment of mutation status across multiple locations of the colon of 10 patients, and describe one case where at least 3 separate *TP53* mutations arise in non-neoplastic colon, each of which gives rise to neoplastic lesions several years later, with the most recently detected *TP53* mutant spreading over 3 years to involve the entire length of the colon. Similarly, Salk et al. (17, 18) used mutations of hypermutable polyguanine repeats as surrogate markers of clonal populations, and demonstrate a greater number of clonal populations in patients with concomitant high grade dysplasia (HGD) or CRC. Again, some of the clonal patches from wherein a cancer arose involve a large surface area of the colon, thereby providing further evidence for a “pre-cancerised” colonic epithelial field. Finally, Lai et al. (19) used comparative genomic hybridization to show that chromosomal instability in phenotypically normal mucosa increases with proximity to dysplasia and cancer. While chromosomal instability could be detected in regions extending more than 160 cm of the colon, the authors described clear evidence of clonal mosaicism, with copy number alterations varying greatly even between biopsies less than 2 cm apart.

## Common selective pressures in the development of inflammatory bowel disease and colorectal cancer

IBD is now recognized as a disease of multifactorial etiology, occurring in genetically predisposed individuals, and triggered by as-yet poorly defined environmental insults that generate an aberrant immune response toward an altered gut microbiome (20). One explanation for the increased neoplasia risk in IBD may be the shared etiological factors implicated in both IBD and colorectal carcinogenesis.

### Shared environmental triggers

The rising IBD incidence worldwide over the last four decades has coincided with profound changes in diet and the adoption of a “Western” lifestyle. In terms of recognized environmental risk factors, both IBD and CRC are associated with sedentary populations that consume a poorly balanced diet, consisting of low plant fiber intake and excess consumption of processed red meat (21–23). A whole host of other lifestyle changes are also potentially implicated in both IBD and CRC, including the ingestion of emulsifiers (24, 25) that are found ubiquitously in processed foods, and low vitamin D levels (26, 27) associated with reduced dietary intake and sunlight exposure. The underlying mechanisms are diverse and are poorly understood, and it remains unclear whether the subsequent metabolome, microbiome and epithelial changes generated by a “Western” lifestyle simply represent associated but non-causative downstream effects of overall modern lifestyle habits, if colon neoplasia itself exerts pro-proliferative changes by modifying the luminal microenvironment, or vice versa.

One interesting commonality can be gleaned through the altered fecal metabolomic profile of both IBD and CRC, most notably the reduction of short chain fatty acids including butyrate and propionate, which are by-products of the bacterial fermentation of dietary fiber (28). Butyrate in particular plays a key role in the maintenance of intestinal homeostasis, namely as the preferred energy source of colonic epithelial cells (29). Butyrate exerts an anti-inflammatory impact both directly on resident innate immune cells (30, 31) and colonocytes (32), and indirectly through the maintenance of epithelial barrier integrity (33, 34) and subsequent reduction in bacterial translocation. Moreover, butyrate has also been shown to have anti-neoplastic properties in CRC cell lines, through the modulation of canonical Wnt signaling (35) and inhibition of histone deacetylation (36). This finding has been corroborated in murine models of IBD-CRC, where germ-free mice subjected to chemically-induced inflammation were partially protected from dysplasia and CRC formation when given high fiber diets with prior gut colonization by butyrate-producing *Butyrivibrio fibrisolvens* (37). No benefits were seen from a high fiber diet alone, *B. fibrisolvens* colonization alone, or a high fiber diet combined with colonization by a mutant strain of *B. fibrisolvens* incapable of producing significant quantities of butyrate. *In vitro* studies have demonstrated the antagonistic effect of bacterial fecal sulfides, which are related to consumption levels of red meat and preservatives (38), on butyrate metabolism in colonocytes (39). These findings are corroborated by clinical observations in obesity, in IBD and in colorectal neoplasia. A high fat and/or low fiber diet are known to reduce fecal butyrate levels (40). Patients with active IBD demonstrate lower levels of fecal butyrate (41) and butyrate-producing bacterial species (42). Similarly, while healthy individuals had a higher intake of dietary fiber compared to patients with advanced sporadic colonic neoplasia (43), it is interesting to note that even the subgroup of patients with neoplasia and high fiber intake had a lower level of fecal butyrate and butyrate-producing bacteria compared to matched healthy controls.

### Shared microbiome alterations

A driving role for the microbiome in the development of colitis-associated CRC is less clear, but nevertheless, there are a number of parallels between the microbiome changes in CRC and IBD, that extend beyond loss of overall microbiome diversity and reductions in butyrate-producing *Firmicutes* such as *Faecalibacterium prausnitzii* (44–46). Other notable shared changes are the increased mucosal abundance of *Enterobacter faecalis* and *Escherischia coli* [in particular adherent-invasive *E. coli* (47–49)], as well as *Fusobacterium* species (50, 51).

*Fusobacterium nucleatum* expresses unique adhesins that have been shown to promote CRC (but not non-neoplastic) cell line proliferation through modulation of Wnt signaling (52); *Fusobacterium* load within cancer specimens may even have adverse prognostic implications for patients with CRC (53). In practice, current assessments of species diversity may not fully capture the CRC-relevant microbiome changes in IBD: for example, *Fusobacterium nucleatum* is not only isolated more frequently in patients with IBD, but those isolates from inflamed IBD tissue show greater invasive potential than isolates from non-inflamed tissue of the same patient (54). Early evidence for the association of specific microbiome components with the earliest genomic changes seen in IBD-CRC comes from recent epigenetic studies, where *Fusobacterium* colonization is associated with pro-carcinogenic methylation changes in the non-neoplastic colonic mucosa of 86 patients; this association remained highly significant (OR 16.2, *p* = 0.01) in a multivariate analysis that included recognized clinical risk factors such as age, duration of disease and surrogate markers of inflammation severity (55). Nevertheless, it is remains unclear whether these microbiome changes represent a secondary consequence of disruption in the integrity and function of the epithelial barrier seen in inflammation and neoplasia (56), or are in fact truly independent drivers of inflammation and carcinogenesis.

Studies of adherent-invasive *E. coli* in IL10^−/−^ IBD murine models demonstrate the potential complexity of the relationship between inflammation, the microbiome and carcinogenesis: inflammation does not promote the survival of adherent-invasive *E. coli*, but rather disrupts hitherto poorly defined processes for the natural negative selection of *Enterobacteriaceae* such as *E. Coli* (57). Adherent-invasive *E. coli* in turn drives colorectal carcinogenesis independently of inflammation activity. Interestingly, deletion of the *E. coli* gene coding for the toxin colibactin abrogates this increased cancer risk in IL10^−/−^ IBD mouse models (58), whereas wild type mice colonized with colibactin-producing *E. coli* do not have an increased cancer risk. The carcinogenic effects of colibactin are therefore evident only in the setting of “exposed” epithelium as seen in IBD. *Enterobacter faecalis* on the other hand, while sharing the ability of adherent-invasive *E. Coli* to persist within innate immune cells (59, 60), has been shown to promote carcinogenesis by a more indirect mechanism. Murine macrophages colonized by *E. faecalis* are polarized toward a pro-inflammatory M1 phenotype. These M1 macrophages in turn produce 4-hydroxynonenal, an alkenal by-product of prostaglandin breakdown by cyclo-oxygenase 2 (COX-2) that disrupts mitotic spindle function in colonic epithelial cells, thereby inducing chromosomal instability and phenotypic transformation (61). Collectively, these findings confirm the ability of the microbiome to exert both direct and indirect carcinogenic effects on colonic epithelium in the context of compromised mucosal barrier integrity.

### Immune co-evolution in IBD-associated carcinogenesis remains poorly understood

Active IBD generates a well-characterized cascade of pro-carcinogenic inflammatory changes [see (62, 63) for reviews]. These include but are not limited to TNF-α, which promotes tumor proliferation and invasion through macrophage recruitment and angiogenesis (64), as well as IL-6 and IL-22, with their pro-proliferative, anti-apoptotic effects mediated by epithelial STAT3 signaling (65, 66). As discussed in further detail in the rest of the review, it is clear that the altered immune microenvironment of IBD can have a direct (evolutionary selective) influence on the epithelial cells, by favoring the survival and expansion of some mutant clones over other mutants or non-mutant cells. However, the trajectory of epithelium-microenvironment co-evolution during carcinogenesis, and in particular the dynamic changes in the resident immune system's simultaneous host-protecting and tumor-promoting roles (67), remains poorly understood.

Current evolutionary approaches in carcinogenesis remain very “cancer cell” focused. In one revealing study, Galon et al. have demonstrated that simple measurements of CD3 and CD8 cell infiltration in the tumor core and invasive margin (68) of sporadic CRCs are superior to clinical TNM staging (69) and genetic microsatellite instability status (70) in predicting patient prognosis. The aberrant immune function that is characteristic of IBD may manifest itself in cancer immune-epithelium co-evolution: for example, IBD-CRC demonstrates greater lymphocyte infiltration compared to sporadic CRC, but with no associated prognostic improvement (71). This may be explained by impaired lymphocyte cytotoxicity in IBD, as reflected by reduced cancer cell apoptosis and lower granzyme B expression in lymphocytes infiltrating IBD-CRC compared to sporadic CRC.

Changes in the mucosal immune cell composition and function that may occur *prior to* malignant transformation remain poorly characterized. A competent immune system plays a clear role in the surveillance for neoplastic growth and the elimination of entire neoplastic lesions. IBD low grade dysplasia (LGD) is thought to regress in the majority of patients (72), regression of sporadic colonic adenomas has also been demonstrated (73), and a role for immune clearance is feasible in both scenarios. Indeed, murine studies provide useful insight into potential immunoediting mechanisms at the earliest stages of neoplastic formation; for example, the transfer of CD4+ CD25+ T_reg_ cells from wild type mice into APC^Min/+^ mice reduces adenoma burden in an IL-10 dependent fashion (74).

A key finding in IBD carcinogenesis is that a significant proportion of IBD LGD lesions demonstrate a level of aneuploidy comparable to that of established CRCs (75); even normal IBD epithelium can bear significant chromosomal copy number alterations (76). Aneuploidy is associated with mutations in genes involved in the DNA damage response (77); indeed *TP53* is the classical initiating mutation in IBD-CRC. Aneuploid cells are normally subjected to negative selection in healthy tissue because of the significant associated proteotoxic, metabolic and replicative stressors (78); therefore this finding in IBD suggests that certain patterns of copy number alterations increase fitness, such that these added “costs” are outweighed by cell-specific “benefits” within the context of the colitic microenvironment, such as less restricted replication and immune evasion. Along this line of thought, it is noteworthy that pan-cancer analyses demonstrate that increased tumor aneuploidy is associated with reduced expression of cytotoxic immune markers, M2 polarization of macrophages, and increased cell cycling and proliferation (77). The mechanisms for these alterations are unclear and the authors speculate that aneuploidy may also impair certain aspects of MHC class I antigen presentation, thereby promoting immune evasion. Evidence to support this hypothesis comes from studies of human lung and breast cancer xenografts in mice, demonstrating how aneuploid cells subvert lethal epithelial responses triggered by cytoplasmic translocation of DNA during mis-segregated mitosis (79) despite upregulation of various inflammatory responses, including the cGAS-STING pathway, which evolved to combat viral infection by detection of extranuclear DNA (80). In aneuploid cancers, activation of the cGAS-STING pathway does not generate the expected downstream canonical NFκB and type I interferon signaling, but rather drives the non-canonical NFκB signaling cascade more typically seen in myeloid-derived cells, which the authors speculate may represent a form of immune mimicry (79).

MHC class II molecules also play an important role in colonic neoplastic change. Normally restricted to traditional antigen presenting cells, MHC class II expression can be induced in transformed epithelial cells, increasing in frequency during the adenoma-carcinoma transition, with a corresponding increase in the density of tumor-infiltrating lymphocytes (81). Moreover, metastasis is associated with a loss of MHC class II expression across multiple solid cancers including CRC (81–83). An example of the role of MHC molecules in colitis-associated carcinogenesis is given by a case-control study of patients with ulcerative colitis, which demonstrates that HLA-DR17 expression (a particular serotype recognizing HLA molecule) is associated with increased CRC risk and methylation-induced HLA-DR silencing, while HLA-DR7 and HLA-DQ5 are associated with reduced CRC risk (84). The authors speculate that these patients may be particularly sensitive to the oncogenic effects of the altered microbiome in IBD (84); indeed MHC class II polymorphisms are known to be associated with susceptibility to infection-mediated cancers such as *Helicobacter pylori* associated gastric adenocarcinoma and HPV-associated cervical squamous cell cancer.

### Shared genetic predisposition

In practice, the evidence for a shared genetic predisposition to both IBD and colorectal cancer remains very limited. In fact, a GWAS study comparing the 181 most common IBD susceptibility variants with those known to predispose to CRC in the general population demonstrate only one shared variant (rs11676348, which lies immediately upstream of *CXCR2*), that actually increases UC risk while *lowering* CRC risk (85). We note that there have been no GWAS studies within IBD patients that identify the subset of patients most at risk of developing dysplasia and cancer, and potentially “classical” GWAS for CRC risk may have included some IBD CRC cases. Nevertheless, these overall findings emphasize the greater importance of acquired (and in particular environmental) factors as the driver for both conditions, which (unlike genetic predisposition) are modifiable, and may therefore be targeted to alter the course of tumor evolution at its earliest stages for cancer prevention.

## Patients with IBD provide an “ideal” human model for the evolutionary study of colorectal carcinogenesis

IBD patients undergoing endoscopic surveillance offer an ideal human system for an evolutionary approach to studying the time course of colorectal carcinogenesis over a patient's lifetime. First, routinely collected biopsies at colonoscopy, years before a cancer is detected, form a rich tissue archive that is amenable to temporospatial evolutionary analysis. Similarly, the metachronous and synchronous nature of IBD neoplastic lesions provides another serendipitous opportunity for the assessment of clonal relationship between lesions separated by time and/or space. Finally, patients with IBD-CRC routinely undergo a complete resection of their colon and rectum rather than a limited segmental resection, thereby allowing for the detailed mapping of mutant populations in phenotypically normal colonic epithelium both proximal to and distal from a neoplasm (75), and facilitating correlation with local microenvironmental factors.

### The relapsing-remitting nature of inflammatory bowel disease drives epithelial clonal evolution

In the following sections, we discuss the significant body of evidence (14) demonstrating that the recurrent cycles of ulceration and healing typical of IBD generate evolutionary pressures that:

- Increase the rates of epithelial stem cell mutations within crypts through more rapid cell cycling,
- Promote the survival of mutant clones that can tolerate an inflammatory environment during periods of disease activity, and
- Allow the expansion of mutant clones that can more rapidly repopulate the healing mucosa.

### The intestinal crypt as the evolutionary unit of the colon

The adult colon is lined by approximately 10 million crypts, which are single cell layer invaginations of the colon lining composed of 1,000–4,000 columnar epithelial cells (86), centered around an estimated five to seven rapidly cycling LGR5-positive stem cells at the crypt base (87–90). In mice, stem cells divide roughly daily (90); their progeny become differentiated as they migrate from the crypt base to the luminal surface over a period of 5–7 days (91) before their shedding into the intestinal lumen. In humans, the dynamics are less well quantified, but are likely a little slower (88). Any mutations that persist in the crypt must therefore have first arisen within a long-lived stem cell. As a result, disparate crypts, bearing stem cells with potentially differing stochastically accumulated mutations, can independently evolve in response to localized microenvironmental pressures; these pressures can vary significantly across the length of the colon (92). For mutant clone fixation to occur within the entire crypt, the “founder” mutant stem cell must first replace all other crypt base stem cells via symmetric divisions in a process referred to as niche succession (93). For those mutations that provide no survival advantage, niche succession through neutral drift within the human intestinal crypt is a thought to be a slow stochastic process that is due to low mutation rates and slow stem cell loss/replacement rates under homeostatic conditions, although reported rates remain under debate (88, 89). Stem cells bearing classical driver mutations for CRC subvert this process (89), with accelerated growth dynamics and likelihood of niche succession due to increased fitness advantages supplied by oncogenic mutations. The colonic crypt stem cells from which a future cancer arises are thought to have already acquired approximately half the somatic mutational burden of the future tumor prior to malignant transformation (94).

Lateral mutant clonal spread in the colon is driven by crypt fission (95) and/or crypt regeneration (96), the processes by which crypts grow. Crypt fission represents a key homeostatic mechanism that heals the mucosa in response to ulceration or other injury (97). Under normal conditions, colonic crypt fission is a surprisingly infrequent event, with fewer than 1% of all crypts dividing in a single year (89).

### IBD activity provides a survival advantage for mutant clones that can survive in a hostile inflammatory environment

Evidence for the importance of the inflammatory microenvironmental context in selecting for crypt stem cells harboring key pro-oncogenic mutations comes from lineage tracing experiments in recombinant mouse models (98). Under normal conditions, stem cells bearing *TP53* mutations are no more likely to replace their neighboring wild type stem cells within the crypt base, while *APC* and *KRAS* mutations, which are more frequently encountered in sporadic CRC (75), offer a clear survival/growth advantage. However, in the context of chemically-induced colitis, the relative fitness of *TP53* mutant stem cells increases, as demonstrated by a 58% probability of mutant niche succession 21 days after colitis induction. This finding is consistent with studies on human IBD tissue showing that *TP53* mutations and loss of heterozygosity occur at a much higher frequency in IBD-CRC compared to sporadic CRC, and are an early event in IBD-driven carcinogenesis that can even be detected in non-dysplastic mucosa (75, 99, 100). This contrasts to sporadic colonic neoplasia, where *TP53* mutations are typically reported to be a late event in the adenoma-carcinoma sequence (101).

*TP53* plays a critical role in inducing cell cycle arrest, senescence or apoptosis in cells with damaged genomes, as well as in cells exposed to severe metabolic and oxidative stressors (102) typically generated by severe IBD flares. On the other hand, colonic epithelial cells in the non-inflamed colon are not exposed to such a hostile microenvironment until the final stages of the adenoma-carcinoma transition, therefore the evolutionary pressure selecting for *TP53* mutations arises much later in sporadic CRC (103). Interestingly, *TP53* mutations are also able to generate an inflammatory response in their own right through oncogenic “gain-of-function” effects (104): human organoid studies and *in vivo* studies in p53^mut/+^ mice demonstrate that epithelial cells bearing a *p53* mutation commonly observed in IBD (R273H) can exacerbate their local inflammatory microenvironment, by prolonging TNFα-induced NFκB activation, eventually generating flat dysplastic lesions with secondary cancers typical of those seen in IBD (105). Therefore, the early *TP53* mutations encountered in IBD may in fact act as independent drivers of IBD carcinogenesis through their downstream microenvironmental effects.

Oncogenic mutations that provide stem cells with a survival advantage within a crypt will often accelerate crypt fission as well. For example, data from murine (106) and human (89) studies confirm that the classical *KRAS* mutation seen in sporadic and to a lesser extent in IBD-associated neoplasia (G12D) increases crypt fission rates by at least one order of magnitude; murine studies also confirm accelerated crypt fission rates following *p53* mutation (107).

### Colorectal carcinogenesis in IBD is driven by accelerated cell turnover and “premature” colonic aging

Studies using Ki67, a cellular marker expressed during the active phases of the cell cycle, demonstrate an expanded proliferative zone in crypts from both regenerating IBD epithelium and early dysplasia when compared to normal colonic crypts (108). The precise stem cell population responsible for regenerating the human colonic epithelium in IBD is uncertain, with evidence from murine studies suggesting dynamic contributions during the inflammation and regeneration phases. LGR5+ expression drops dramatically during the acute stages of colitis but increases dramatically during regeneration (109); other studies confirm that LGR5+ expressing stem cells may be very sensitive to intestinal injury (110) but nonetheless crucial to crypt regeneration (111). Inflammation has been shown to recruit long-lived and hitherto quiescent DCLK1+ tuft cells (112) from the crypt wall (113) that can acquire stem cell properties in the absence of LGR5+ stem cells, reconstituting the entire crypt, including the LGR5+ stem cell niche. Interestingly, these DCLK1+ cells did not proliferate or initiate neoplastic progression following conditional APC knockdown without the addition of an inflammatory stimulus (113).

It is thought that a rapid increase in stem cell numbers and subsequent clustering within the crypt base may act as a trigger for the initiation of crypt fission (114, 115). These observations may explain why active IBD, with an expanded stem cell proliferative zone, is associated with a crypt fission rate that is 30- to 70-fold higher than that of uninflamed mucosa (116), further accelerating cell turnover and clonal expansion.

These shifts in the properties of the stem cell niche in the context of injury and inflammation are in part driven by stromal cells that generate the necessary canonical and non-canonical Wnt signaling molecules necessary for epithelial reconstitution and subsequent maintenance of homeostasis (96). In mouse models, these stromal cells responsible for Wnt signaling have recently been identified as telocytes (117). Induction of acute colitis in mice by DSS ingestion results in an expansion of GLI1+ expressing telocytes (118). Of interest, a coding polymorphism in human *GLI1* that generates a variant protein with reduced transactivation is associated with predisposition to ulcerative colitis in populations of Northern European descent (119), further highlighting the role inflammation-modulated Wnt signaling in IBD pathogenesis. In addition, changes in the luminal metabolic and microbiome micro-environment induced by colitis may also play a role in promoting expansion of the stem cell niche. For example, intestinal intraluminal butyrate inhibits the proliferation of LGR5+ stem cells *in vitro*, and is thought to play a role in confining the stem cell niche to the crypt base, where differentiated colonocytes on the crypt walls have consumed the butyrate as their primary energy source (120). Reductions in butyrate associated with the microbiome dysbiosis of active IBD may therefore allow stem cells in injured crypts that are more directly exposed to luminal contents to continue dividing, thereby contributing to the accelerated cell turnover.

There now exists a substantial body of evidence demonstrating that the predominant mechanism for carcinogenesis in IBD is accelerated cell turnover and rapid colonic “aging,” and that IBD-CRC is *not* a consequence of direct DNA damage from reactive oxygen and nitrogen species as traditionally thought (121). First, trinucleotide context mutational signature analysis (122) of IBD-CRC demonstrates a preponderance of C>T substitutions at NpCpG trinucleotides (123) (mutational signature 1) in keeping with aging driven by rapid cell cycling (124), with no detection of signatures typical of direct DNA exposure to a genotoxic environment (signatures 4, 7, 11, 22, 24, and 29). Second, multiple studies report telomere shortening, another surrogate marker of accelerated aging, in the colitic mucosa (18, 125–127). Finally, patients with ulcerative colitis bearing HGD or cancer demonstrate significant aging-related CpG island hypermethylation signatures in colitic mucosa far from the site of the neoplasia; these changes were not seen in UC mucosa of neoplasia-free patients or healthy controls (128).

The aforementioned finding of colonic aging through rapid cell cycling, combined with the heterogeneity of somatic mutations seen in IBD-associated CRCs (123, 129, 130), limits the extent to which any single animal model can replicate colitis-associated neoplasia formation (131). AOM-DSS mice remain the most utilized model, with mice exposed to the carcinogen azoxymethane (AOM), followed by repeated ingestion of dextran sulfate sodium (DSS) to induce inflammation. However, whole exome sequencing of these mouse cancers shows little overlap in terms of mutational landscape with human CRC, in particular the near absence of the most common IBD-CRC driver mutations such as *TP53, APC, KRAS*, and *PIK3CA*, no shared (132). Indeed, AOM-DSS mouse CRC are striking for the over-representation of C>T substitutions, which is more typical of DNA damage by alkylating agents like azoxymethane (132). Likewise, IL10^−/−^ mice develop colitis-associated cancers that do not demonstrate the chromosomal instability (133) that is typically encountered in most human IBD-CRCs (134); recent studies suggest the need for additional microbial and immunological stressors to improve IL10^−/−^ mouse model fidelity (61). In conclusion, the inability of current mouse models to replicate the diversity and dynamic shifts of the human microbiome, nor the cumulative effect of chronic inflammation and aging generated by colitis and time in patients, implies that assessment of IBD carcinogenesis requires study of the underlying process in patient-derived samples.

## Evolutionary biomarkers: a novel approach merging biology and mathematics

The stochastic nature of evolutionary changes (e.g., mutation accumulation, clonal expansion and selection) requires adequate time (years) for carcinogenesis to progress normal cell phenotype to malignancy in the human body. Using mathematical models, we can formulate mathematical expressions describing these evolutionary changes, and use the expressions to relate these evolutionary parameters to age-dependent epidemiological cancer incidence curves (135). In addition to the chronological age of the patient as an initial biomarker of CRC risk, biological aging of the colitic bowel itself may be considered as a potential marker of progression to neoplasia due to its prominence in the pathogenesis of IBD carcinogenesis explained above. The concept is based on measuring both the extent and speed of genetic evolution as a proxy of how “close to cancer” the cells have become (136). Tissue age is difficult to measure *in vivo*, but can be estimated with computational methods like Bayesian inference that have been used in molecular clocks applied to somatic epigenetic (137) and genomic (138) data from non-dysplastic Barrett's esophagus, a precursor of esophageal adenocarcinoma that is characterized by intestinal metaplasia, driven by chronic acid- induced inflammation (139).

Partly due to accelerated cell turnover, IBD-CRCs show genomic diversity, both in terms of the inter-tumor permutations of genomic alterations leading to cancer formation, as well as the intra-tumor genomic heterogeneity in established cancers. IBD-CRCs may share only some of the same driver mutations (75), and some premalignant colonic adenomas do not have a detectable driver mutation in any of the 20 most frequent driver genes (140). For this reason, biomarkers of cancer risk that assay one molecular alteration or a single pathway will likely never be sensitive or specific enough to justify routine clinical use in cancer surveillance recommendations for patients with IBD.

Along with tissue age, other measures of *evolvability* may provide a more reliable marker of cancer risk in which the issue of intra-patient heterogeneity may be circumvented for clinical management. For example, IBD patients at risk of developing cancer may demonstrate high diversity in clonal composition that continues to change over time, while low risk patients may harbor few, if any, mutant clonal populations with size distributions that remain stable over time (14). A cornerstone principle of cancer evolution is that genomic diversity acts as the substrate for natural selection in the inflamed colonic bowel; the more diverse the colonic epithelial cell population, the more likely a well-adapted, “dangerous” clone will be present, outcompete other clones, and evolve toward a malignant phenotype. This concept of evolutionary biomarkers, defined in terms of ecological diversity measures, has been repeatedly demonstrated to be predictive of neoplastic progression in patients with Barrett's esophagus (138, 141, 142).

Rather than seeking a set pathway of necessary changes from IBD to IBD-CRC, we can quantify measures of evolvability that are “agnostic” to any specific oncological pathway, by capturing a range of distinct molecular processes that may be potentially driving an individual patient's cancer formation. By assaying the genomic alteration burden from spatially distributed biopsies, a wide range of evolutionary measures can be generated. For example, we could provide evidence of clonal sweeps by identifying shared genomic alterations across multiple individual biopsies, implying that they originated from a common founder. Similarly, clonal mosaicism can be assayed spatially between biopsies by measuring the genomic “distance” between the somatic mutations in these biopsies (where genomic distance is measured by the number of divergent mutations between two biopsies).

**Figure 2:**
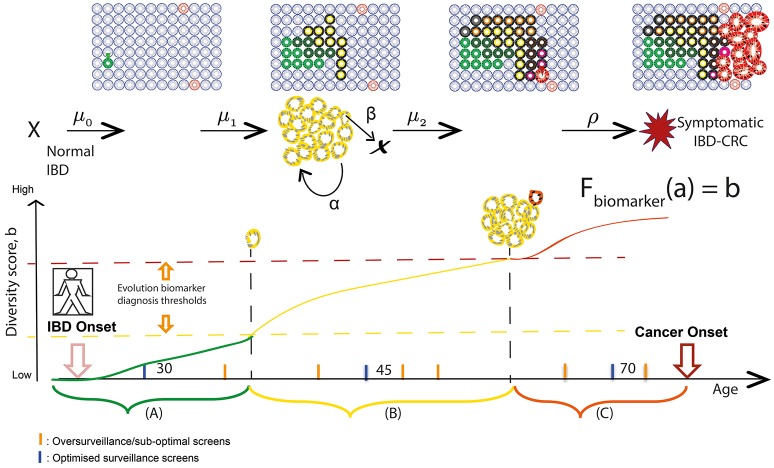
Screening and surveillance intervals in IBD using an evolutionary approach. Patients in a population develop IBD at a known age; the aim for prevention is to provide a baseline endoscopic screen before some of those patients will have developed neoplasia. Hypothetical “windows of opportunities” for surveillance quantify timescales during which **(A)** pre-initiated cells gain alterations (early diagnosis), **(B)** initiated clones expand (promotion) and become more genetically diverse (early intervention), and **(C)** preclinical malignant clones evolve (progression) and could be thwarted (early detection). Levels of diversity increase in an evolving inflamed microenvironment and may act as a biomarker of evolutionary trajectory position. We aim to relate biomarker levels (b) measured during surveillance screens at specified ages (a) to multistage model parameters via variables in a theoretical function F_biomarker_. As a theoretical example, if the relationship between diversity and size of premalignant clone (yellow) is known, then we can measure the rate of growth of the clone during surveillance and predict at what age the clone will be a certain size and likely harbor a detectable phenotype change of preclinical malignancy.

Finally, due to the extensive sampling of the IBD colon during routine surveillance, all assays can be measured over time in the same patient, with chronological rates of change potentially providing another measure of “evolvability.” A simple measure of growth is the difference between the clone size at two time points (which in its coarsest resolution, could be represented by the number of equidistant biopsies bearing that clone) divided by the difference in time points of endoscopic screening examinations for precursor lesions in IBD. In this way, we can utilize the lifelong surveillance of IBD patients to collect the genomic measurements for more precise calculations of IBD-specific evolutionary parameters such as initiation (μ), birth (α), and death (β) rates of mutant clones in individual patients (Figure 2).

Indeed, temporal studies are vital when defining a “window of opportunity” for clinical intervention—i.e., when early pre-cancerous development may be observed/removed and further, when cancer risk can be reliably predicted (143, 144). Progression to IBD-CRC is driven by a series of rate-limiting evolutionary events (such as *TP53* mutation and critical copy number changes as described earlier in the review), while ongoing accumulation of heterogeneous genomic alterations occurs via genetic drift in crypts throughout patient lifetime. These salient rate-limiting events governing IBD evolution, which are not clearly understood but could also be assayed using a combination of particular mutations and evolutionary biomarkers, serve as the boundaries between windows of screening opportunity and may be reflective of histopathological stage. An evolutionary approach to IBD surveillance would aim to identify periods during a patient's lifetime when highly evolved and aberrant clones would be detectable during surveillance, and when an early intervention would be beneficial, in order to tailor surveillance screens accordingly. This method of monitoring patient-specific rates of evolutionary change (such as increasing levels of genetic diversity in clones) can be used to predict the age(s) at which clones will attain a threshold size and/or diversity (see Figure 2). We can then use this information to make dynamic, personalized recommendations for the most efficient next-surveillance-screen time; this approach of theoretically predicting biomarker value change in the future has been demonstrated in mathematical models for *Helicobacter pylori* driven gastric cancers (145).

Thus far, the modeling done in studies of premalignant risk stratification in Barrett's esophagus is typically standard survival analysis where the evolutionary biomarker (such as level of genetic diversity) is used as the predictor variable. More sophisticated models attempt to infer the temporal dynamics of clonal evolution, and extrapolate inferred trajectories to predict future disease state [for example, inference of time-dependent evolution model parameters for clone growth in Barrett's esophagus (138)].

Mathematical modeling of carcinogenesis in IBD is in its infancy. Several types of mathematical modeling approaches may be useful to incorporate measures of the evolutionary process into temporospatial models of IBD cancer evolution for calibration and prediction of patient-specific trajectories. Agent-based models for clone growth of the evolutionary process *in silico* can employ simulations of patient-specific parameters (such as the rate of clonal expansion of a particular clone in a particular individual, or the patient-specific mutation rate) and explore the effect of spatial tissue constraints and microenvironmental changes on disease progression. Such models require extensive and detailed biological data at the outset for parameterisation, though, meaning that they may be impractical to apply in practice. Continuum models of growth may be used to predict general tissue change that can evaluate the effects of spatial sampling bias (e.g., quadrant biopsy tissue removal) on detection of rare subclones, but the increased abstraction of these modeling formulations necessitates that some biological detail is neglected.

Population level models can also be used to incorporate epidemiological data (e.g., cancer incidence, premalignant prevalence) and thus describe cancer evolution in a population with IBD. An example would be multistage clonal expansion models, a family of cell-based stochastic models positing that cancer is caused by the accumulation of rare events that define the boundaries of the initiation-promotion-progression stages of carcinogenesis (135). This theoretical framework integrates time-varying risk factors into the analysis of cancer epidemiological data (such as incidence and multifocal sizes of pre-malignant lesions), wherein stages from normal to malignant transformation are defined by the occurrence of rate-limiting events (e.g., *TP53* mutation). Finally, hybrid models can combine the above techniques that are calibrated to multiple levels of data [see reference (137) for an example of modeling Barrett's esophagus clinical screening using agent-based tissue sampling *in silico* combined with cellular parameters calibrated to esophageal adenocarcinoma population data]. Choosing the appropriate model (and model type) is non-trivial, and depends upon the utility that is sought from the model.

By using the equations of the multistage mathematical model with estimated parameter values, we can then define such windows by solving for the probabilities (analytically and numerically) that an individual will most likely harbor a premalignant or malignant lesion of a screen-detectable size at endoscopic screening/diagnosis, and then use the outcome of each screen to benchmark the progress of evolution and iteratively predict the next window to recommend surveillance screen times.

At present, such temporospatial information about clonal evolution needed in these mathematical model predictions is generally lacking, and so consequently is not used in the design of IBD surveillance protocols. Candidate molecular markers that have been associated with progression in IBD (described earlier in the review) including aneuploidy, methylation assays, microsatellite instability and mutational panels of key driver genes in IBD-CRC (such as *TP53, APC, KRAS*, and *CDK2NA*). Of these, only aneuploidy, as measured using flow cytometry, has been shown to date to carry prognostic potential in IBD (76, 146, 147). Dynamical information using one or more of these markers could potentially enhance clinical practice beyond current *ad hoc* screening interval recommendations, which are based on crude clinical features (4, 5).

## From colitis to cancer: translational implications of utilizing an evolutionary approach

Pathogenic genomic alterations (e.g., point mutations and chromosomal copy number alterations) are known to occur in phenotypically normal epithelium many years before a cancer forms (16, 75, 146, 148). Enumeration of these mutations may aid in risk stratifying patients who will more likely progress to IBD-CRC for more aggressive surveillance and treatment, while reducing surveillance requirements for lower risk patients. By using the aforementioned evolutionary approach, we envisage a more personalized approach to cancer risk assessment that combines patient demographic details and endoscopic features (149) with genomic assays.

At present, a significant proportion of patients with low grade dysplasia are advised to have a complete resection of their colon and rectum in light of the high risk of multifocal neoplasia (150). For these patients, the ability to define the extent of mutant clonal spread can justify a more limited surgical resection, with particular focus on the potential for rectal-sparing surgery, thereby avoiding the need for an ileo-anal pouch or permanent stoma.

A natural extension of this evolutionary approach to IBD carcinogenesis is that altering the inflammatory selection pressure may modify future cancer risk. At present, we are uncertain as to whether standard IBD anti-inflammatory or immunosuppressive therapies can halt (or possibly even reverse) the formation, expansion and/or evolution of mutant clones. 5-aminosalicylates, an anti-inflammatory class of drugs that form the first line of therapy for patients with UC, are thought to reduce the incidence of IBD-associated dysplasia and IBD-CRC (151). The precise mechanism remains unclear and is probably multi-faceted; 5-aminosalicylate use has been shown to reduce inflammation-generated β-catenin signaling in the mid- and upper crypt (152). β-catenin is a key transcription factor in the Wnt pathway, with aberrant constitutive Wnt signaling (through phosphorylation and nuclear translocation of β-catenin) extending beyond the crypt base stem cell being a common initiator in colorectal adenoma and carcinoma formation through the expansion of the crypt stem cell niche. In sporadic adenomas this is achieved through *APC* gene mutation; indeed, similar findings have been noted in APC^Min/+^ mice, where non-steroidal anti-inflammatory drug (NSAID) use selectively increased the apoptosis rate in crypt stem cells with nuclear or phosphorylated β-catenin by over fivefold (153).

In practice, prospective randomized controlled trials that can assess the specific impact of the different medical therapies on IBD dysplasia and CRC risk will be challenging to conduct, and will probably be underpowered due to the required large patient cohort size (to handle inter-patient variability) and long follow-up time. Instead, a proxy for cancer-risk reduction will be the minimization of the cumulative inflammatory burden of the colitic bowel through the achievement of deep remission (154). An understanding of the evolutionary dynamics of carcinogenesis in IBD, and its evaluation *in vivo* in the presence and absence of chronic disease activity, may compensate for any limitations in our understanding of the precise anti-neoplastic mechanisms of anti-inflammatory IBD therapies. Indeed, experimental data on the feasibility of disease-modulating drugs to limit clonal expansion and progression in human tissue has emerged during the study of Barrett's esophagus. In Barrett's esophagus, NSAID use has been shown to modulate clonal evolution (155), with a reduction of both the burden and diversity of functional mutations affecting key cancer-associated pathways compared to matched controls (156).

Quantification of immune-epithelial cell co-evolution is an important area for future research. Both cell populations can be described quantitatively by complex system models (157, 158) with marked plasticity, resulting in a near infinite possible set of permutations (clonal and subclonal populations in the case of cancer, B cell and mucosal T cell receptor repertoire composition combined with microbiome diversity in IBD), and a susceptibility to sustained external selection pressures that can “promote” and “fix” clinically deleterious traits (e.g., loss of response to IBD therapy). As the “selfish” drive of the individual colon cancer cell to expand comes at the expense of the multicellular human host, so the same approach can be used to understand and model the conditions driving the “selfish” activation and/or expansion of aberrant immune cell populations.

Much like traditional cancer chemotherapies, current IBD therapies are the product of a reductionist approach that targets one or several pathways, which are of varying importance between patients (hence the variable and incomplete response rate to IBD therapies), and which have a minor impact on the long term course of the disease [as demonstrated by the persisting need for surgery (159) despite advances in medical therapy]. In cancer, evolutionary adaptive therapies, that aim to control tumor burden while simultaneously limiting the selection pressure driving the emergence of resistant mutant clones, offer a new paradigm in oncological management that already shows promise in pilot clinical trials (160). Similar treatment paradigms are needed in IBD patients: fecal microbiota transplantation shows promise as such an intervention in ulcerative colitis (161), with its concomitant alterations in microbiome diversity and composition. Indeed, fecal microbiota transplantation is most efficacious in patients with more recent diagnoses of ulcerative colitis (162), possibly because this intervention is performed prior to the irreversible “fixation” of aberrant adaptive immune clones in such patients.

## Conclusion

Inflammatory bowel disease represents an ideal model for the study of human cancer using an evolutionary approach. Routine surveillance colonoscopies provide a serendipitous opportunity to observe somatic evolution over space and time *in vivo*. Moreover, somatic evolution is accelerated in IBD through the relapsing-remitting nature of disease flares. Direct and detailed temporospatial assays of clonal populations, together with their co-evolving immune and microbiome components of the mucosal microenvironment, now feasible using the latest sequencing technologies, can be leveraged toward the development of an “evolutionary” biomarker that can better predict an individual patient's cancer risk. Finally, an evolutionary systems approach (163), currently utilized in the study of carcinogenesis, may offer a novel paradigm for understanding the concomitant immunological evolution that is vital for escape from immune surveillance and promotion of tumor growth.

## Author contributions

IA and KC contributed to the writing of this review. TG supervised the writing of this review.

### Conflict of interest statement

The authors declare that the research was conducted in the absence of any commercial or financial relationships that could be construed as a potential conflict of interest.
